# Sero-Molecular Markers and Genetic Diversity of Hepatitis B Virus Isolated From Hemodialysis Patients From Jenin District, West Bank, Palestine

**DOI:** 10.1155/cjid/6981644

**Published:** 2025-09-19

**Authors:** Kamal Dumaidi, Amer AL-Jawabreh, Areej Zraiqi, Jana Zaid, Suhair Ereqat, Nabeel Salami, Abedelmajeed Nasereddin

**Affiliations:** ^1^Department of Medical Laboratory Sciences, Arab American University, Jenin, State of Palestine; ^2^Leishmaniasis Research Unit, Jericho, State of Palestine; ^3^Biochemistry and Molecular Biology Department, Al-Quds University, Abu Deis, East Jerusalem, State of Palestine; ^4^Clinical Laboratory Department, Dr. Khalil Suleiman Governmental Hospital, Jenin, State of Palestine; ^5^Al-Quds Bard College, Al-Quds University, Abu Deis, East Jerusalem, State of Palestine

**Keywords:** haplotype networking, hepatitis B virus (HBV), occult hepatitis B virus infection (OBI), phylogenetic analysis, whole genome sequence (WGS)

## Abstract

**Background:** Hepatitis B virus (HBV) infection remains a major global health challenge, especially among high-risk groups such as hemodialysis (HD) patients.

**Aim:** This study investigated the prevalence of sero-molecular markers and the genetic diversity of HBV in 160 Palestinian HD patients. Blood samples were tested for HBV serological markers (HBsAg, anti-HBc, and anti-HBs) and screened using nested PCR. Whole genome sequencing was conducted on PCR-positive samples to identify HBV genotypes and subgenotypes.

**Results:** The overall HBV prevalence among HD patients was 3.75%, comprising 1.9% with overt infection (HBsAg +ve) and 1.9% with occult HBV infection (OBI). HCV was detected in 1.9% of patients. Evidence of past exposure (anti-HBc positive) was observed in 20% of patients, and 45% showed serological immunity with anti-HBs levels ≥ 10 IU/mL. Although the values of the genetic diversity estimators such as K, S, *η*, and *π* were approximately as twice as those for the S-region, the S-region produced a more reasonable phylogenetic tree and haplotype networking but under the condition of accurate sequencing and adequate number of investigated sequences. Phylogenetic trees and haplotype networking of the WGS and S-region revealed a clustering pattern based on genotypes and subgenotypes with two Palestinian WGS clustering in Subgenotype D1, while the other two in Subgenotype D3. Genetic diversity analysis revealed high haplotype diversity (Hd) (0.98–1.00) with high h:n ratio (0.9–1.00) and low nucleotide diversity (*π*) (0.007–0.027) indicating slight variation between any two given sequences. This is explained by purifying selection, recent population expansion, or constrained evolution as neutrality test values such as Tajima's D were negative (−0.5 to −1.86).

**Conclusion:** HBV infection remains prevalent among HD patients, including both overt and occult forms. Genotype *D*, specifically Subgenotypes D1 and D3, predominates in the study population. The HBV S-region is a sufficient surrogate for population genetics investigations.

## 1. Introduction

Despite the availability of an effective vaccine, hepatitis B virus (HBV) infection remains a major global health burden and the leading cause of viral hepatitis. In 2015, viral hepatitis accounted for approximately 1.34 million deaths worldwide, with HBV-related complications responsible for 60% of these fatalities. Notably, HBV was associated with 39% of liver cancer deaths and 29% of cirrhosis-related mortality. The global prevalence of HBV infection in the general population is estimated at approximately 3.5%, corresponding to over 250 million individuals living with chronic HBV infection worldwide [1].

HBV is an enveloped virus with a partially double-stranded, relaxed circular DNA genome approximately 3200 nucleotides in length. It belongs to the family Hepadnaviridae [2]. HBV is traditionally classified into 10 genotypes (A–J) based on ≥ 8% nucleotide divergence across the whole genome and/or ≥ 4% divergence in the S gene, which encodes the hepatitis B surface antigen (HBsAg) [3]. More recently, comprehensive phylogenetic analysis of over 4000 HBV sequences has refined this classification, confirming the existence of 10 genotypes (A–J) and identifying 24 distinct subgenotypes, including A1–A3, B1–B5, C1–C6, D1–D6, and F1–F4 [4].

The global public health strategy to combat viral hepatitis, endorsed by 194 WHO Member States, aims to reduce hepatitis-related mortality by 65% and new infections by 90% by the year 2030, using 2015 as the baseline. This strategy emphasizes the delivery of comprehensive services, including vaccination (particularly the HBV vaccine), screening, antiviral treatment, and long-term care for chronic infection. However, despite progress and the imminent target year, HBV prevalence remains notably high, especially among high-risk populations such as hemodialysis (HD) patients. Adding to the complexity of elimination efforts is the emergence of occult hepatitis B virus infection (OBI), a form of infection characterized by the presence of HBV DNA in the absence of detectable HBsAg [1].

A recent systematic review and meta-analysis reported an overall HBV prevalence of 7.32% among HD patients, with substantial variation across geographic regions. The highest prevalence was observed in South America (9.37%), followed by Oceania (8.45%), Asia (7.44%), Europe (7.07%), Africa (5.52%), and North America (4.32%) [5]. The elevated prevalence of HBV in this population is largely attributed to both direct transmission via contaminated dialysis equipment and indirect transmission through healthcare personnel. The nature of the dialysis procedure characterized by repeated vascular access and close contact with blood and medical instrument, further increases the risk of HBV transmission, especially in settings where infection control practices are inadequate [5]. Additionally, numerous studies have demonstrated suboptimal immunogenicity of the HBV vaccine in patients with end-stage renal disease (ESRD), primarily due to impaired immune function. This reduced responsiveness places ESRD patients at heightened risk for HBV infection, even after standard vaccination protocols, thereby necessitating enhanced preventive strategies and possibly modified immunization regimens [6]. An additional challenge in the management of HBV infection, particularly in high-risk settings such as HD units, is the emergence of OBI. This covert form of infection presents a substantial risk for undetected HBV transmission, especially in dialysis environments where repeated blood exposure is common. The typically low or fluctuating viral loads associated with OBI can evade standard screening protocols, thereby undermining infection control efforts. Moreover, OBI has been implicated in the progression of chronic liver disease, including cirrhosis and hepatocellular carcinoma, highlighting its importance as a significant clinical and public health concern [7, 8]. Therefore, the aim of this study was to investigate the prevalence of serological and molecular markers of HBV infection among HD patients attending HD units in Jenin governmental hospital, located in the northern West Bank, Palestine. In addition, complete genome sequencing was conducted to characterize the genetic diversity and determine the genotypes and subgenotypes of the isolated HBV strains.

## 2. Materials and Methods

This cross-sectional study included 160 HD patients who were randomly selected from the HD unit at Jenin Governmental Hospital during the period of January–December 2023. Demographic information and clinical history were collected using a structured questionnaire administered during patient interviews, supplemented by a review of medical records. Ethical approval for the study was obtained from the Palestinian Ministry of Health (Reference No.: 162/1481/2022), which authorized the use of anonymized clinical samples and patient data. Verbal informed consent, though not documented, was obtained in accordance with the minimal-risk nature of the study and the use of fully anonymized data.

### 2.1. Blood Sample

Before the initiation of HD, the leftover portions of the blood samples collected by the HD staff during the routine dialysis investigation were obtained and distributed into two tubes. The first tube was used for serological detection of HBV markers, including HBsAg, anti-HBc, and anti-HBs, while the second tube was used for molecular analyses such as DNA extraction, PCR amplification, and sequencing.

### 2.2. Serology Assays

Serum samples from each patient were analyzed using commercial immunoenzymatic assays to detect HBV serological markers, including “HBsAg” (one Version ULTRA, REF: SAG ULTR. CE), hepatitis B core antibody total “anti-HBc” (HBcAb, REF: BCAB. CE) and “anti-HBs” (REF: SAB.CE). Furthermore, hepatitis C virus (HCV) antibodies were tested using the HCV Ab kit (REF: CVAB.CE.96). All immunoenzymatic assays were performed according to the manufacturer's instructions. All commercially used immunoenzymatic kits were obtained from Dia-Pro Diagnostic Bioprobes Srl, Italy, and are reported to have 100% sensitivity and specificity.

### 2.3. DNA Molecular Assay

#### 2.3.1. DNA Extraction

Two hundred microliter of the serum from each sample was used for viral DNA extraction using the QIAamp Viral DNA/RNA Extraction Kit (Qiagen, Hilden, Germany) in accordance with the manufacturer's instructions.

#### 2.3.2. PCR

The HBV-DNA was amplified using nested PCR with two primer sets targeting the viral polymerase gene, as described by Selabe et al. [2]. The products from the second PCR round were analyzed by electrophoresis on 2% agarose gels stained with ethidium bromide. A 647-bp band was considered a positive result. Negative (nuclease-free water) and positive controls were included in each PCR assay.

#### 2.3.3. Viral Whole Genome Amplification and DNA Library Preparation

This study utilized two previous studies primers to amplify HBV whole genome and included newly designed primers from this study, as shown in Table 1 [3, 4]. The design of new primer sets came following amplification failure of at least one product from each primer set described in the two aforementioned studies which could have resulted from the length of the target amplicon (> 1 kbp) and low viral load of the tested samples. For primer design, the primers from the two aforementioned studies were mapped on the HBV genome, NCBI Reference Sequence GenBank accession number JN664911 and designed new shorter PCR targets in order to increase the PCR sensitivity. The best amplified product based on fragment size and intensity of the PCR band (Supporting Figure 1) was used in two separate pools to avoid primer overlaps, PCR reaction of Sets 1, 3, 5, and 7 were done in multiplex PCR as Pool 1, while PCR primers 2, 4, and 6 were mixed together in the second multiplex PCR, then the 2 PCR products were pooled in one tube, then purified with 1X AMPureXP beads (Beckman Coulter, Brea, CA, USA), and quantified by Qubit Fluorometer DNA assay (Thermo Fisher Scientific, Waltham, MA, USA). DNA libraries were prepared, using the Illumina DNA Prep Kit (Illumina, San Diego, CA, USA) according to the manufacturer's recommended protocol. All samples' libraries were pooled together, and DNA libraries were sequenced with a NextSeq 500/550 Mid Output Kit v2.5 (150 Cycles) on NextSeq 500 machine (Illumina, San Diego, CA, USA).

#### 2.3.4. Viral Whole Genome Assembly

Binary Base Call (BCL) files as output from the NextSeq 500 sequencing machine were converted to FASTQ format using BCL to FASTQ command line (bcl2fastq v2.20.0.422 Copyright (c) 2007–2017 Illumina, Inc.). FASTQ files were analyzed using the galaxy program (Galaxy Version 0.7.17.1) (https://usegalaxy.eu/) [5]. HBV consensus sequences were obtained by mapping reads with BWA-MEM-map medium and long reads (> 100 bp) against the HBV (strain ayw) genome, NCBI Reference Sequence: NC_003977.2. The mapping was displayed with a local Integrative Genomics Viewer (IGV) and the consensus sequence from the IGV was copied and accepted as the sequence of each sample. The IGV results were confirmed by using Geneious 8.0.5. HBV genotyping and subgenotyping were carried out using the NCBI genotypic tool (https://www.ncbi.nlm.nih.gov/projects/genotyping/formpage.cgi).

### 2.4. Statistical Analysis

Degree of association between HBV infection and demographic and laboratory parameters was estimated by calculating the odds ratio, Fisher's exact test at a *p* value < 0.05 by using EpiInfo free software. Maximum likelihood (ML) phylogenetic 1000-iteration bootstrap consensus trees were constructed for HBV whole genome sequence (WGS) and S-region using MEGA-X software [6]. The phylogenetic trees were edited using iTOL (https://itol.embl.de) [7]. A haplotype analysis network based on single nucleotide variation (SNV) was built based on median-joining method using PopART 1.7 software [9] with zero epsilon as a default parameter. Nexus format file generated by DnaSP Version 6.12.03 was used as an input file for PopART 1.7 software. Coloring of the haplotype network was based on the country of origin of HBV DNA sequences. The genetic diversity estimators, population differentiation, and neutrality tests were calculated using DnaSP Ver. 6.12.03 [10]. The meg file produced by MEGA-X was used as an input file for DnaSP Ver. 6.12.03.

## 3. Results

### 3.1. Study Population Characteristics

The overall prevalence of HBV among HD patients was 3.75% (6/160) (3 patients “1.9%” had overt HBV infection [HBsAg positive], and 3 “1.9%” had OBI). All of them were male, yet insignificant despite the low *p* value (*p*=0.03) and high OR (OR = 11.87) due to overlapping of the OR = 1.0 where the value 1.0 is included in the 95% CI (CI = 0.65–214.3). Moreover, 1.9% of the samples (3/160) were positive for HCV but significantly less in HBV HD patient (OR = 75.5, CI = 5.6–1015, *p* value = 0.003). The median age of the patients was 54 years ranging from 20 to 85 years, with 82.2% of patients over 40 years. The male-to-female ratio was 1:1.16. Additionally, 84% of the HD patients had received blood and/or blood products, however without any significant difference (OR = 2.66, CI = 0.15–48.8, *p* value = 0.59). The level of liver enzymes was not significantly different between HBV patients and nondiseased (Table 2). Of the patients tested, 66 (45%) demonstrated baseline immunity to HBV, as indicated by anti-HBs levels ≥ 10 IU/mL; nevertheless, immunity status did not significantly differ between the HBV and non-HBV patients (OR = 0.81, CI = 0.16–4.1, *p* value = 1.0). All patients, except two, reported receiving the full HBV vaccine doses. Furthermore, 32 out of 158 patients (20%) tested positive for anti-HBc which was shown to be significantly different between HBV and non-HBV (OR = 8.86, CI = 1.5–50.8, *p* value = 0.016). All including the six patients reported receiving the full three-dose HBV vaccination; however, 9 of the 32 patients (27%) did not show protective immunity levels (anti-HBs < 10 IU/mL), suggesting prior exposure to HBV infection.

Table 3 summarizes the demographic characteristics and sero-molecular markers of hepatitis B-positive cases. It includes data on age, sex, hepatitis B markers, PCR results, and vaccination status for each patient. The table provides a detailed overview of the serological profiles and PCR outcomes, illustrating various combinations of markers such as HBsAg, anti-HBc, and anti-HBs, with all patients consistently exhibiting positive PCR results.

### 3.2. Whole Genome Sequencing

Seven primer sets were used to sequence HBV whole genome (Table 1). Primer Set 4 (forward and reverse) was exclusively designed by this study, while Sets 2, 3, 5, 6, and 7 were partially modified with either the forward or the reverse primers redesigned. Four samples were successfully sequenced for HBV whole genome. The four genomes were deposited in the GeneBank under the Accession numbers: PQ772197.1, PQ772198.1, PQ772199.1, and PQ772200.1.

### 3.3. Phylogenetic Analysis

**Figure 1:**
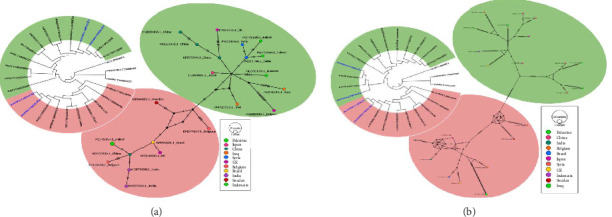
(a) Maximum likelihood (ML) phylogenetic tree of the S-region of 23 studied DNA sequences of the Subgenotypes D1 and D3 that contained the four Palestinian sequences. The analysis was based on the Tamura–Nei model [8] with 1000 bootstrap value shown. Tree was constructed by MEGA-X(6). (b) Median-joining haplotype networking of the S-region. Each node represents a haplotype. Node colors represent the country of origin of isolate, as indicated in the legend. The diameter of the node circle is proportional to the number of sequences. The number of hatch marks on the connecting lines between nodes indicates the number of mutations between nodes. The clusters in the trees and the networks are represented by the colors red and green. (b) Maximum likelihood (ML) phylogenetic tree of the same characteristics, but using the WGS of 23 studied DNA sequences.

The phylogenetic analysis was studied for 79 HBV DNA sequences randomly selected from the GenBank that covered the range of HBV genotypes from A to H. The two analyses included constructing ML phylogenetic trees using both the whole genome (2989 nt) and the S-region (835 nt) (Supporting Figures 2 and 3). The ML phylogenetic tree constructed from the WGSs produced five clusters, which largely corresponded to the known HBV genotypes and, furthermore, Genotype D subclustered into four according to Subgenotypes D1, D2, D3, D4, D5, and D7 (Supporting Figure 2) with Subgenotypes D4/D7 having shorter branch distances indicating less divergence and closer common ancestry. Subgenotypes D1/D2 are to a lesser extent showing the same pattern as in D4/D7. The Genotypes F, G, and H fell into the same cluster with shorter branches between them. In contrast, the phylogenetic tree based on the S-region exhibited the same overall cluster pattern but demonstrated greater resolution, yielding eight clusters with enhanced discriminatory power as it separated Genotype G into a separate cluster (Supporting Figure 3). Additionally, one Genotype G sequence (AB625343.1) was observed to cluster with Genotype F, suggesting a potential of misclassification. Phylogenetic trees based on neighbor-joining (NJ) showed similar clustering patterns as did the ML trees (data not shown). The four Palestinian HBV whole genomes grouped in D1 and D3 subclusters were further analyzed for phylogenetic trees, haplotype networking, and genetic diversity parameters. The analyses included 23 DNA sequences including the Palestinian sequences. The ML phylogenetic trees for the whole genome and the S-region were fully congruent with each other as both yielded two clusters which were based on the subgenotypes (D1 and D3) (Figures 1(a) and 1(b)). The four Palestinian HBV sequences were split equally in the two clusters, D1 and D3 in both trees. Cluster I (D1) included 13 DNA sequences with the two Palestinian DNA sequences PQ772199.1 and PQ772200.1, while Cluster II (D3) contained 10 DNA sequences including the other two Palestinian DNA sequences, PQ772197.1 and PQ772198.1. The phylogenetic tree clusters were supported by the haplotype networking which produced two haplogroups in both the whole genome and the S-region but more explicit when using the S-region (Figures 1(a) and 1(b)).

### 3.4. Genetic Diversity

The genetic diversity parameters for the HBV-WGS and HBV–S-region disclosed two clusters, I (D1) and II (D3) (Table 4). The percentage of mutations in the WGS is 16% (485/2989) compared to 8.5% (71/835) for the S-region. In total and per cluster, the genetic diversity (*π*) for the WGS is twice as high as the S-region. The percentage of the average number of nucleotide differences between two randomly chosen sequences from within in the WGS population (K) were as twice (2%) as those chosen from the S-region (1%). The percentage of the number of segregating sites (S) as a major polymorphism indicator is higher for the HBV whole genome (14%) than the (S) of the HBV S-region (8%). The haplotype diversity (Hd), total, and per cluster are the same between the WGS and S-region. In both WGS and S-region, the Hd was high, while genetic diversity (*π*) is low. The two Palestinian DNA sequences in Cluster I (PQ772199.1 and PQ772200.1) have a high degree of similarity as evidenced by low number of variable (polymorphic) sites (*S* = 3), low genetic diversity (*π* = 0.0036 ± 0.001), and low number of mutations (Eta = 3). At the same time, the neutrality indexes showed deviation from neutrality (negative value) with varying significance levels for Tajima's D and Fu-Li's F tests for the WGS, except for Tajima's D for Cluster II of the WGS (Tajima's *D* = −0.68 and Fu-Li's F −0.50). However, for the S-region Cluster I significantly deviated from neutrality, Tajima's D (−1.89), and Fu-Li's F (−2.50) and so as for the total S-region (−2.77). In general, for the WGS and S-region or between the clusters within, the Hd was high, genetic diversity statistics (*π*) was low, and neutrality tests were negative (Table 4). The recombination parameter (R) based on the variance of the average number of the nucleotide difference between pairs of DNA sequences is considered intermediate (*∞* > *R* > 0) that ranged from 2.1 to 56.0.

Based on Wright's grading system, both targets showed high (> 0.25) Fst with medium (0.25–0.99) Nm values [16]. Furthermore, the Kxy, the average ratio of nucleotide differences between populations, is high supporting population differentiation (Table 5). In contrast, the other interpopulation estimators (Dxy, Gst, and Da) that are functions of nucleotide substitution and haplotype frequency were low (Table 5).

## 4. Discussion

Over a decade ago, our research team reported that approximately 22.7% of HD patients in northern Palestine were infected with the HBV, with 8.2% as overt HBV infection and 12.5% with OBI [19]. This elevated prevalence at that time highlighted the urgent need for stringent infection control practices within HD units to reduce the risk of HBV transmission including the implementation of infection prevention protocols and continuous, standardized HBV screening. These prevention measures likely contributed to the observed decline in HBV prevalence, at 3.75% among HD patients in the present study. Globally, the prevalence of HBV among HD patients varies considerably. For instance, a study conducted in Tocantins, Brazil, reported a significant decline in HBV prevalence, from 4% in 2001 to 0.8% in 2014-2015. In Syria, a multicenter study conducted in Damascus found a prevalence of 3.2%, while in Ghana, the rate was 7.7%. In contrast, a recent study from the Ismailia governorate in Egypt reported a notably high prevalence of 19% among HD patients [20–25]. In addition, a global systematic review and meta-analysis revealed regional variations in the prevalence of HBV among HD patients. The review reported a prevalence of 7.44% in Asia, 4.32% in North America, 7.07% in Europe, 5.52% in Africa, 8.45% in Oceania, and 9.73% in South America. These findings underscore the global burden of HBV in HD populations and highlight the need for region-specific infection control strategies [26]. The observed variations in HBV prevalence across different countries and regions can be attributed to several factors, including the implementation of infection control protocols, the effectiveness of global measures to prevent the spread of blood-borne viruses, local epidemiological conditions, and diagnostic methodologies employed. The present study's findings confirm a significant decline in HBV prevalence among HD patients, highlighting the effectiveness of infection prevention strategies.

This study showed that among the six HBV cases, three were classified as OBI. The three OBI cases were positive for anti-HBc and, notably, two of which also tested positive for anti-HBs. The prevalence of OBI has shown to be internationally variable, with reported prevalence ranging from 0.11% in Japan to as high as 30% in Iran [27, 28]. These considerable differences in prevalence may be explained by differences in the sensitivity of the molecular detection methods and case definition, whether based on anti-HBc–positive cases or on all HD cases as denominators for estimation.

In this study, 20% of HD patients tested positive for anti-HBc, indicating significant exposure to HBV. Comparable findings have been reported in countries such as Brazil (12%), Iran (26.3%), Ghana (12.5%), and Yemen (47.5%) [17, 20, 25, 26]. These differences likely stem from varying HBV prevalence, infection control standards, and diagnostic methods. The consistently elevated anti-HBc rates in HD patients underscore the need for improved infection prevention measures in dialysis settings.

The observed immune protection rate in our study indicates that only 45% of HD patients vaccinated against HBV achieved anti-HBs levels ≥ 10 IU/mL, the protective threshold. This finding is consistent with the recent reports from other regions, which have documented seroconversion rates of 50.7% in Ghana, 30% in Brazil, and 66.3% in Iran [21, 24, 29]. Tong et al. demonstrated that in immunocompetent individuals, long-term protection against hepatitis B is primarily mediated by immune memory, with protective immunity generally indicated by anti-HBs concentrations ≥ 10 IU/mL following vaccination [30]. In contrast, immunocompromised individuals such as those undergoing HD often fail to sustain durable immune memory, and their protection against HBV relies predominantly on the continuous presence of circulating anti-HBs antibodies [30]. Another study has shown that HD patients with postvaccination anti-HBs levels between 10 and 100 mIU/mL often experience a decline in protective antibody titers within 1 year [31]. Furthermore, Ghadiani et al. demonstrated that even with vaccination, the seroconversion rate in HD patients remains significantly lower compared to the general population [32]. Consequently, the low observed protection rates against HBV among HD patients emphasize the critical importance of regular monitoring of anti-HBs titers and the timely administration of booster doses to sustain adequate immunity in this high-risk group.

In the present study, the ML and NJ phylogenetic analyses based on WGS and S-region showed that the four WGS-sequenced HBV isolates were classified as Genotype D and Subgenotypes D1 and D3. These findings are consistent with previous studies from Palestine, which also reported a predominance of Genotype D, with Subgenotype D1 accounting for approximately 90% of cases. Genotype D is recognized as the most prevalent HBV genotype in the Mediterranean region and the Middle East [25, 33–35]. Within the D genotype, Subgenotype D1 is the most frequently reported in our region. In contrast, Subgenotype D3 has been documented in several countries, including Egypt, Iran, and Brazil [36–38].

In this study, neither the phylogenetic tree nor the haplotype networking analysis of both WGS and S-region showed any particular clustering pattern pertaining to the geographical origin, rather the clusters were based on genotypes and subgenotypes of the HBV (Supporting Figures 2 and 3). This finding was supported by HBV phylogenetic studies elsewhere targeting the whole genome or the S-region whether based on NJ or ML methods which have elucidated the presence of clusters based on genotype or subgenotypes without any effect relation to the geographical origin [37, 39–41]. However, wider scale studies related the genotypes and subgenotypes of HBV to geographical distribution [42–45]. The phylogenetic trees (ML and NJ) constructed using a large dataset of 79 HBV DNA sequences revealed greater resolution when the S-region was analyzed, yielding eight clusters with multiple distinct subclusters, compared to five clusters identified through whole genome sequencing. These findings are in line with the recent phylogenetic studies that highlight the enhanced discriminatory power of S-region–based phylogenetic analysis for HBV genotype and subgenotype classification, relative to WGS [34, 46]. One plausible explanation for this discrepancy between WGS and S-region is the tolerance of missing data by the larger target (whole genome) compared to the smaller target (S-region). The intolerance of the S-region to missing data deviated sequences to fall from one cluster into another. The DNA sequence that deviated from Genotype G into Genotype F cluster was due to missing 8% (65 nt) inherited from sequencing failure. Error-free sequencing plays a pivotal role in genetic population studies especially when the investigated DNA target is small, while it can be tolerated if the whole genome was under investigation. This was evidenced when the properly sequenced D1 and D3 clusters containing the four Palestinian sequences were analyzed with different models (ML and NJ) and different targets (WGS and S-region), yet generating a perfect match phylogenetic tree. The haplotype networking of the S-region was clearer with less hash marks that represent SNVs and less small black circles that indicate missing individuals as compared to the whole genome haplotype networking which contained excessive numbers of hash marks and black circles brought about by the larger size of WGSs. Accurate WGSs with sufficient number of investigated individuals (sequences) may result in a reasonably complete haplotype networking.

As for the genetic diversity and neutrality analyses, the two study targets, whole genome and S-region, and their respective clusters have shown high Hd values (0.98–1.00) with high h:n ratio values (0.9–1) and at the same time low genetic diversity (*π*) values (0.007–0.027) (Table 4). These findings suggest that the majority of DNA sequences are identical with slight variation between any two given DNA sequences indicating low degree of polymorphism with many closely related haplotypes in the population but insufficient to exhibit real genetic diversity. This indicates that the HBV population may have experienced a very recent rapid population expansion which is supported by negative Tajima's D (−0.5 to −1.86) and Fu-Li's F test (−0.5 to −2.77) with Cluster I (D1) being significantly (*p*-value < 0.05) deviated from neutrality in both regions, the whole genome and the S-regions. Such rapid expansion and growth of HBV population have introduced new rare mutations as indicated by the high percentage of the total number of mutations in WGS (16%) and the S-region (10%) which is considered higher than the usual percentages, 8% and 4%, respectively [47]. In addition, the low genetic variation is upheld by the low percentage of the average number of nucleotide differences between two randomly chosen sequences from within the population in the HBV whole genome (*K* = 2%) and the HBV S-region (*K* = 1%). Similarly, the percentage of variable segregating sites (S) in the WGS is 14% compared to 10% in the S-region. Phan et al. studied the whole genome for 930 HBV WGSs and found that the percentage of the number of polymorphic segregating sites (S) to be 19.6% which is higher than that of this study [40]. Contrary to this, the same study showed that the average number of nucleotide differences (K) was 21, which are lower than that of this study (84) [40]. A possible explanation for the low genetic variation in HBV may lie in the compact structure of the virus itself with four major overlapping genes and the replication mechanism that resembles RNA viruses. The compact structure of HBV leads to what has been called ‘constrained evolution' that imposed a lower substitution rate per nucleotide site than in RNA viruses which eventually led to lower genetic diversity among the HBV population [48, 49].

The recombination parameter (R), which depends on the population size and recombination rate per sequence, is neither zero (absence of recombination) nor infinite (*∞*) (free recombination), but in between. The genetic recombination is higher in the S-region (*R* = 29.9) than in the whole genome (*R* = 11.7) (Table 4). An increase in R (> 0) elevates the likelihood of genetic recombination via horizontal DNA transfer between HBV genotypes under coinfection, thereby driving the generation of novel haplotypes and accelerating genetic diversification, population expansion, evolutionary bottlenecks, and signatures of recent positive selection [50–53]. The interpopulation differentiation parameters showed high Fst (0.39) in the two targets, whole genome and S-region. However, the Nm values were not low (0.0–0.249) as expected but rather intermediate (0.39) indicating that population differentiation could have happened without or little gene flow (migration) involving transfer of DNA from one population to the other, intrapopulation genetic drift, and genetic selection. The medium level of gene flow (Nm) supports genetic differentiation between Clusters I (D1) and II (D3) in both targets, WGS and S-region (Table 5). The main limitations of the present study are its single-center design, missing clinical and demographic data for some patients, and a small number of HBV-positive cases, which may reduce statistical power. Additionally, vaccination status was partly reported, with incomplete documentation of the final dose. Another limitation which could have affected the statistical analysis is the low prevalence of OHB in HD patients which is 3.7% in our study. The low prevalence leads to a wide 95% CI range (Table 2) that had a negative effect on the precision level of significance.

## 5. Conclusion

The S-region of HBV forms an adequate surrogate for population genetics studies in setups where WGS is beyond the reach. The significantly high rate of OBI among the HD population represents a critical challenge that must be considered in the formulation and implementation of HBV eradication strategies. Furthermore, using primer sets that amplify shorter fragments offers a cost-effective, sensitive, and scalable approach for HBV whole-genome sequencing, especially in low viral load samples.

## Figures and Tables

**Table 1:** The seven sets of primers used in the study for amplifying HBV whole genome showing the original primers and the newly designed primers designated as ‘This study'.

PCR	Oligo name	Oligo sequence	Product (bp)	Primer binding position	Gene binding sites	Reference
1	PCR2Pol1b	GGACTATCAAGGTATGTT	416	448–465	Polymerase (P) genes	[3]
	PCR1Pol2AS	TAACCCCATCTTTTTGTTTT		844–863		[3]

2	HBV_PCRF_D	CAAGTGTTTGCTGACGCAAC	525	1176–1156	P, S genes	This study
	PCR2P5AS	CCTCAAGGTCGGTCGTTGAC		1682–1701		[3]

3	PCR3HB7ES	TGGAGACCACCGTGAACGCC	840	1609–1628	Precore, polymerase-reverse transcriptase (Pol), PreS1 (PreS1), and X protein (X) genes	[3]
	HBV_PCR_R_D	CCCAGTAAAGTTCCCCACCT		2470–2490		This study

4	N_2344F	GTTAGACGACGAGGCAGGTC	624	2365–2385	Precore, polymerase-reverse transcriptase (Pol), PreS1 (PreS1), and X protein (X) genes	This study
	N_2968R	AGGTGTCCTTGTTGGGATTG		2969–2989		This study

5	PCR3P2S	TCACCATATTCTTGGGAACAAGA	570	61–80	Gene “C” core capsid protein, gene = “X,” and polymerase (P) gene	[3]
	N_208R	AGAAAAACCCCGCCTGTAAC		611–631		This study

6	N_191F	TTCCTAGGACCCCTTCTCGT	443	175–195	Gene = “X” and polymerase (P) gene	This study
	AR	GATGATGGGATGGGAATACARGTG		595–618		[4]

7	N_715F	CCCACTGTTTGGCTTTCAGT	568	718–738	Polymerase (P) gene	This study
	BR	GCWAGGAGTTCCGCAGTATGG		1266–1286		[4]

**Table 2:** The degree of association between demographic and laboratory characteristics and HBV infection among hemodialysis patients.

Variable	Total	HBV	Non-HBV	OR (95% CI)	*p* value (< 0.05)
Sex					
Male	85	6	79	11.87 (0.65–214.3)	0.03
Female	72	0	72		
Total	157	6	151		
Age group (years)					
20–39	27	2	25	2.28 (0.39–13.2)	0.31
≥ 40	118	4	114		
Total	145	6	139		
GOT					
High	93	2	91	0.19 (0.03–1.1)	0.06
Normal	39	4	35		
Total	132	6	125		
GPT					
High	82	3	79	0.58 (0.1–3.0)	0.67
Normal	49	3	46		
Total	131	6	125		
ALP					
High	49	2	47	0.68 (0.12–3.8)	1.0
Normal	68	4	64		
Total	117	6	111		
Transfusion:					
Yes	130	2	124	2.66 (0.14–48.8)	0.59
No	25	4	25		
Total	155	6	149		
Anti-HBsAg					
Anti-HBsAg+	81	3	78	0.81 (0.16–4.1)	1.0
Anti-HBsAg−	66	3	63		
Total	147	6	141		
HCV					
Anit-HCV+	32	4	1	75.5 (5.62–10.2)	0.003
Anti-HCV−	126	2	151		
Total	158	6	152		
Anti-HBc					
Anit-HBc+	3	2	28	8.86 (1.5–50.8)	0.016
Anti-HBc−	155	4	128		
Total	158	6	152		
Immunity status					
Anti-HBs < 10 U/L	81	3	78	0.81 (0.16–4.1)	1.0
Anti-HBs ≥ 10 U/L	66	3	63		

**Table 3:** Demographic characteristic and seromolecular markers of hepatitis B positive cases.

HBV cases	Age/years	Sex	HBsAg	Anti-HBc	Anti-HBs	PCR	HBV	HBV genotype	Vaccinated^∗^
4JHD	43	Male	Negative	Negative	Negative	Positive	OBI	ND	Yes
5JHD	32	Male	Positive	Positive	Negative	Positive	Overt HBV	HBV/D3	Yes
6JHD	32	Male	Positive	Positive	Positive	Positive	Overt HBV	ND	Yes
8JHD	54	Male	Negative	Positive	Positive	Positive	OBI	HBV/D1	Yes
9JHD	72	Male	Positive	Positive	Negative	Positive	Overt HBV	HBV/D1	Yes
10JHD	53	Male	Negative	Negative	Positive	Positive	OBI	HBV/D3	Yes

*Note:* OBI: occult hepatitis B infection.

Abbreviation: ND, not done.

^∗^A full three-dose vaccination was administered as per the Palestinian Ministry of Health vaccination scheme.

**Table 4:** Genetic diversity and neutrality comparison indices between the WGS and S-region of the HBV included in the study between the two genotypes D1 (Cluster I) and Cluster D3 (Cluster II).

	WGS		Total	S-region		Total
	Cluster I	Cluster II		Cluster I	Cluster II	
*L*	—	—	2989	—	—	835
*n*	13.0	10.0	23.0	13.0	10.0	23.0
*h*	13.0	9.0	22.0	13.0	9.0	22.0
*h*:*n* ratio	1.0	0.1	0.96	1.0	0.90	0.96
Eta (*η*)	283	253	485 (16%)	49	23	71 (8%)
Hd ± SD	1.00 ± 0.03	0.98 ± 0.05	0.99 ± 0.014	1.00 ± 0.03	0.98 ± 0.05	0.99 ± 0.014
π ± SD	0.018 ± 0.00	0.024 ± 0.00	0.027 ± 0.00	0.010 ± 0.01	0.007 ± 0.00	0.012 ± 0.00
*K*	55.14	77.14	81.36 (2%)	9.13	6.20	10.39 (1%)
*S*	274	241	448 (14%)	49.0	23	68 (8%)
Tajima's D	−1.82^∗^	−0.68	−1.55	−1.89^∗^	−1.13	−1.79
Fu-Li's F	−2.39^∗^	−0.50	−1.86	−2.50^∗^	−1.08^∗^	−2.77^∗^
*R*	56.0	2.1	11.7	15.6	24.5	29.9

*Note: L*: length of the region/number of sites (#nt), *n*: number of sequences, *h*: Number of haplotypes [11], Hd: haplotype (gene) diversity [11], π: nucleotide diversity (per site) [11], *K*: average number of nucleotide differences between two randomly chosen sequences from within in the population [12], *S*: number of variable/segregating sites [13], Eta (η): total number of mutations [14], and *R*: recombination estimator *R* = 4Nr where *N* is the population size and *r* is the recombination rate per sequence [15]. Tajima's D: relative difference between observed (*π*) and expected genetic diversity [13], Fu Li's F: differences between (η) and (k) [14], ND: not done as the number of sequences in the cluster < 4.

^∗^ *p* < 0.05.

^∗∗^ *p* < 0.01.

**Table 5:** Pairwise population differentiation indices between D1 (Cluster I) and D3 (Cluster II) using WGS and S-region of HBV DNA sequences.

Estimator	WGS	S-region
	I and II	I and II
Fst	0.39	0.39
Nm	0.39	0.39
Kxy	101.81	12.59
Dxy	0.034	0.02
Gst	0.006	0.006
Da	0.013	0.006

*Note:* Fst: Wright's F-statistics, genetic differentiation index as a pairwise genetic distance. Under island model Fst ≈ 1/(4 Nm + 1) [17]. Nm: gene flow and population migration among populations. If diploid under the island model: Nm = (1 − Fst)/4Fst [17]. Kxy: average proportion of nucleotide differences between populations [18]. Dxy: the average number of nucleotide substitutions per site between populations [11]. Da: the number of net nucleotide substitutions per site between populations [11]. Gst: genetic differentiation index based on the frequency of haplotypes [18].

## Data Availability

The data used in this study are available from the corresponding author upon reasonable request.

## Supporting Information

Figure 1: The amplified product based on fragment size and intensity of the PCR band. Detailed analysis of HBV PCR products using the Agilent Technologies 4200 TapeStation system. Figure 2: The maximum likelihood (ML) phylogenetic tree constructed from the whole genome sequences of the HBV genome. A consensus 1000 bootstrap maximum likelihood phylogenetic tree constructed from 79 WGS using MEGA-X. Figure 3: The maximum likelihood (ML) phylogenetic tree constructed from the S-region of the HBV genome. A consensus 1000 bootstrap maximum likelihood phylogenetic tree constructed from 79 S-region using MEGA-X.
