# Supplementary material for: Sero-Molecular Markers and Genetic Diversity of Hepatitis B Virus Isolated From Hemodialysis Patients From Jenin District, West Bank, Palestine
Source: Can J Infect Dis Med Microbiol. 2025 Sep 19;2025:6981644. doi: 10.1155/cjid/6981644 (PMC12473736; doi:10.1155/cjid/6981644)
Supplement: Supporting Information — Additional supporting information can be found online in the Supporting Information section. [file 6981644.f1.zip › Supplementary Figure 3.pdf]

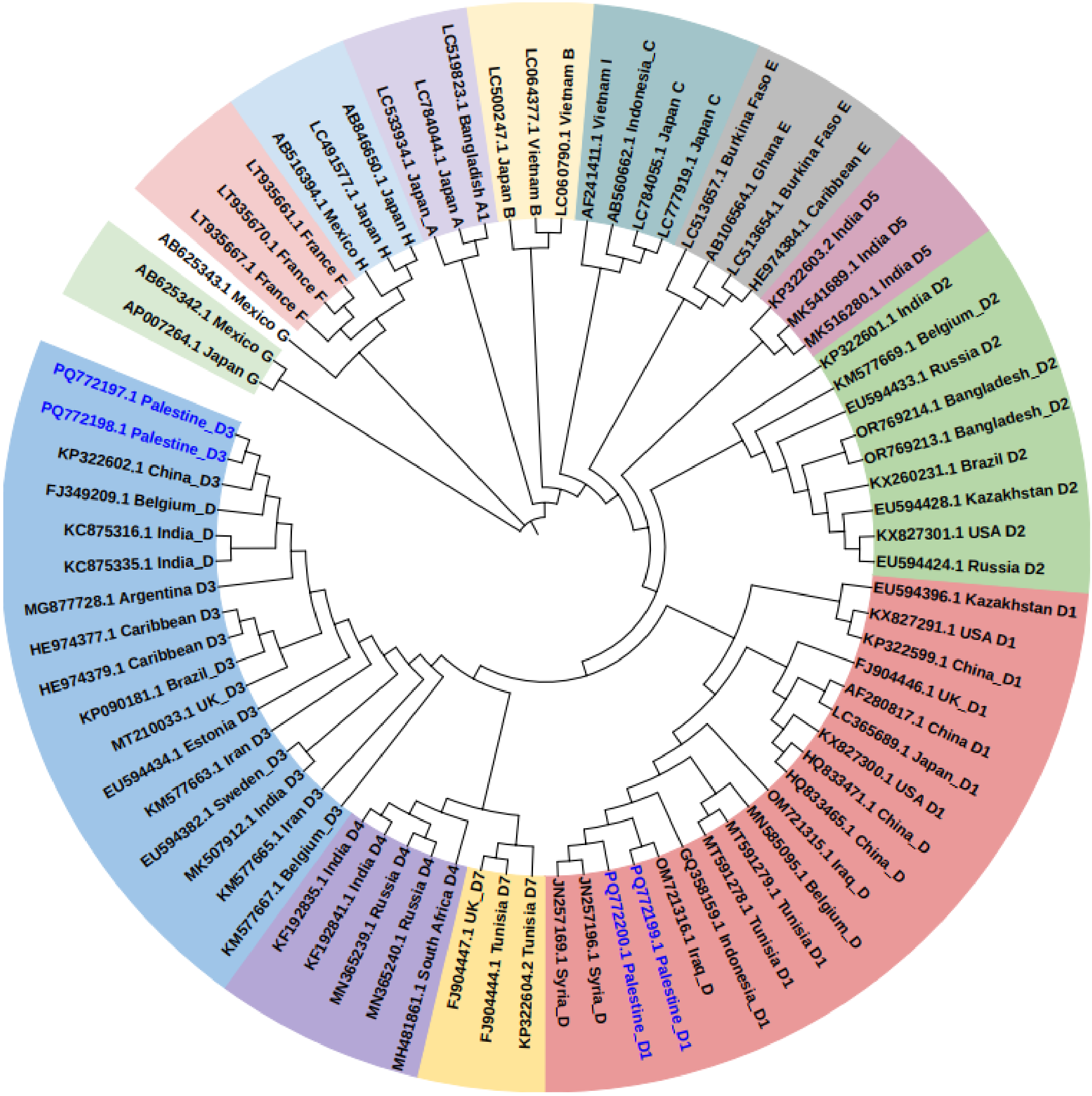

Supplementary Fig 3: A consensus 1000 bootstrap maximum likelihood phylogenetic tree constructed from 79 S-region using MEGA-X.
